# Protective Effects of Intraoperative Nerve Monitoring (IONM) for Recurrent Laryngeal Nerve Injury in Thyroidectomy: Meta-analysis

**DOI:** 10.1038/s41598-018-26219-5

**Published:** 2018-05-17

**Authors:** Binglong Bai, Wuzhen Chen

**Affiliations:** 1 Department of General Surgery (Thyroid Center), Second Affiliated Hospital, School of Medicine, Zhejiang University, Hangzhou, Zhejiang P. R. China; 2 Department of Surgical Oncology, Second Affiliated Hospital, School of Medicine, Zhejiang University, Hangzhou, Zhejiang P. R. China

## Abstract

Recurrent laryngeal nerve (RLN) injury is an intractable complication of thyroidectomy. Intraoperative nerve monitoring (IONM) was designed to prevent RLN injury. However, the results concerning the protective effect of IONM on RLN injury are still controversial. We searched all eligible databases from 1980 to 2017. Meta-analysis was performed to evaluate the effect of IONM on RLN injury. Sensitivity analysis was also conducted to check the stability of our results. There were 34 studies included in the analysis. Overall analysis found a significant decrease in total injury (RR = 0.68, 95%CI: 0.55 to 0.83), transient injury (RR = 0.71, 95%CI: 0.57 to 0.88), and permanent injury (RD = −0.0026, 95%CI: −0.0039 to −0.0012) with IONM. Subgroup analysis found IONM played a preventive role of total, transient and permanent injury in patients undergoing bilateral thyroidectomy. IONM also reduced the incidence of total and transient injury for malignancy cases. Operations with IONM were associated with fewer total and transient RLN injuries in operation volume < 300 NARs per year and fewer total and permanent RLN injuries in operation volume ≥ 300 NARs per year. The application of IONM could reduce the RLN injury of thyroidectomy. Particularly, we recommend routine IONM for use in bilateral operations and malignancy operations.

## Introduction

Recurrent laryngeal nerve (RLN) injury is the most severe complication of thyroid surgery, leading to transient or permanent voice changes, which is one of the most common causes for medical litigation^1^. According to reported data, the incidence of transient RLN injury and permanent RLN injury in thyroidectomy are 2–11% and 0.6–1.6%, respectively^2–4^. In certain situations, the risk of RLN injury is relatively high, including thyroid surgery for malignant tumors, history of neck operation, and surgery of toxic goiter or substernal goiter^5,6^.

Routine visual identification of RLN is applied in thyroid surgery to prevent RLN injury, which serves as a conventional measure to reduce vocal cord paralysis. Dionigi *et al*. found that the causes of RLN injury included transection, clamping, ligatures, suction, traction, thermal injuries and physical compression^7^. Intraoperative nerve monitoring systems (IONM) use the electromyographic signal of vocal muscle movement to reflect the function of the RLN^8,9^. IONM increases the identification rate of RLN, reduces the time of IONM identification and predicts the postoperative function of the vocal cords^10,11^. IONM can be divided into 2 generations, non-continuous intraoperative nerve monitoring (NCIONM) and continuous intraoperative nerve monitoring (CIONM). NCIONM is still the most common method of nerve monitoring, and CIONM is a recent surgical trend that could prevent RLN injury though real-time monitoring.

Although IONM is designed to reduce the incidence of RLN injury, the effect of IONM on RLN injury prevention in thyroid surgery is still controversial^12,13^. Some results found that IONM could reduce transient RLN injury, while other results revealed non-significant help of IONM in RLN injury prevention^4,14,15^. Several meta-analyses have been conducted to discuss this topic^12,13,16–18^. Most of these studies found that IONM was not superior to visual identification in preventing permanent RLN injury^12,18^. Some results indicated that IONM could decrease RLN injury incidence in high risk thyroid surgery, particularly in cancer operations or in those with a previous history of neck operation^17^. For operative sites, incidence of bilateral injury under IONM was relatively higher (0.2%), and staged thyroidectomy was recommended when one-side RLN injury occurred to prevent further bilateral RLN injury^19,20^.

During the last 2 years, many large clinical trials emerged on this topic, which made it necessary to update the data for further conclusion^10,11,21–26^. Considering the existing controversial results and new data, we conducted this meta-analysis to clarify the effects of IONM in thyroidectomy. Subgroup analysis was performed to identify the effects of IONM in bilateral operation, malignancy operation, reoperation group and operation volume.

## Materials and Methods

### Search strategy and selection criteria

We performed a comprehensive literature search in MEDLINE (PubMed), BIOSIS Previews (ISI Web of Knowledge) and Cochrane library from January 1980 to July 2017. We also manually added relevant articles by reviewing the references. Search terms included: “Neuromonitoring” or “nerve monitoring”, “thyroidectomy” or “thyroid surgery”, and “recurrent laryngeal nerve” or “RLN”. Studies included were confined to randomized controlled trials, case control studies and cohort studies, with convertible effects data of IONM in thyroidectomy. If there were several papers extracting from the same population, we would only include the most recent publication. We used the criterion of 6 months to distinguish transient RLN injury from permanent RLN injury, which was consistent with relevant studies^10,27^.

### Data extraction and quality assessment

Data were extracted by two authors (BL Bai and WZ Chen) independently. Detailed information was recorded on first author, year of publication, title, location, study type, diagnosis, inclusion and exclusion criteria, allocation method, study duration, device used, outcome measurement, definition of permanent injury, number of case and control groups, the event of transient and permanent RLN injury, the population type, multicenter research or not, main conclusion and Newcastle-Ottawa Scale (NOS) score. Nerve at risk (NAR) was calculated based on number of lobectomy (1 NAR for each side), subtotal thyroidectomy (2 NARs) and total thyroidectomy (2 NARs). In the section of operation volume, we omitted multicenter research studies for the unavailability of hospital volume data and would discuss the effects of IONM under open surgical operation because of insufficient sample size of endoscopic surgery. The Newcastle-Ottawa Scale for Quality was used to assess the included cohort studies^28^.

### Statistical Methods

Meta-analysis was performed under R environment 3.4.1 through meta package 4.8–3^29^. According to the Cochrane Collaboration, risk ratio (RR), risk difference (RD) and confidence interval (CI) were estimated from the median of the posterior distribution and calculated by the random-effects model with inverse variance weight^30–32^. If zero events occurred in both experimental and control group, the pooled RD estimates and 95% CIs were applied. Otherwise, RR was applied. RR below one or RD below zero indicated a benefit of the experimental intervention. We estimated 95%CI from the 2.5th and 97.5th percentiles of the posterior distribution, and calculated two-sided p values from the posterior distribution. If the 95%CI did not contain the valid value 1, the result was considered to have significant difference. Publication bias was assessed using Begg’s test and Egger’s regression asymmetry test^33^. The heterogeneity between trials was estimated from the median between-trial variance (τ²) observed in the posterior.

### Availability of supporting data

All data are fully available without restriction.

## Results

### Description of studies

**Figure 1:**
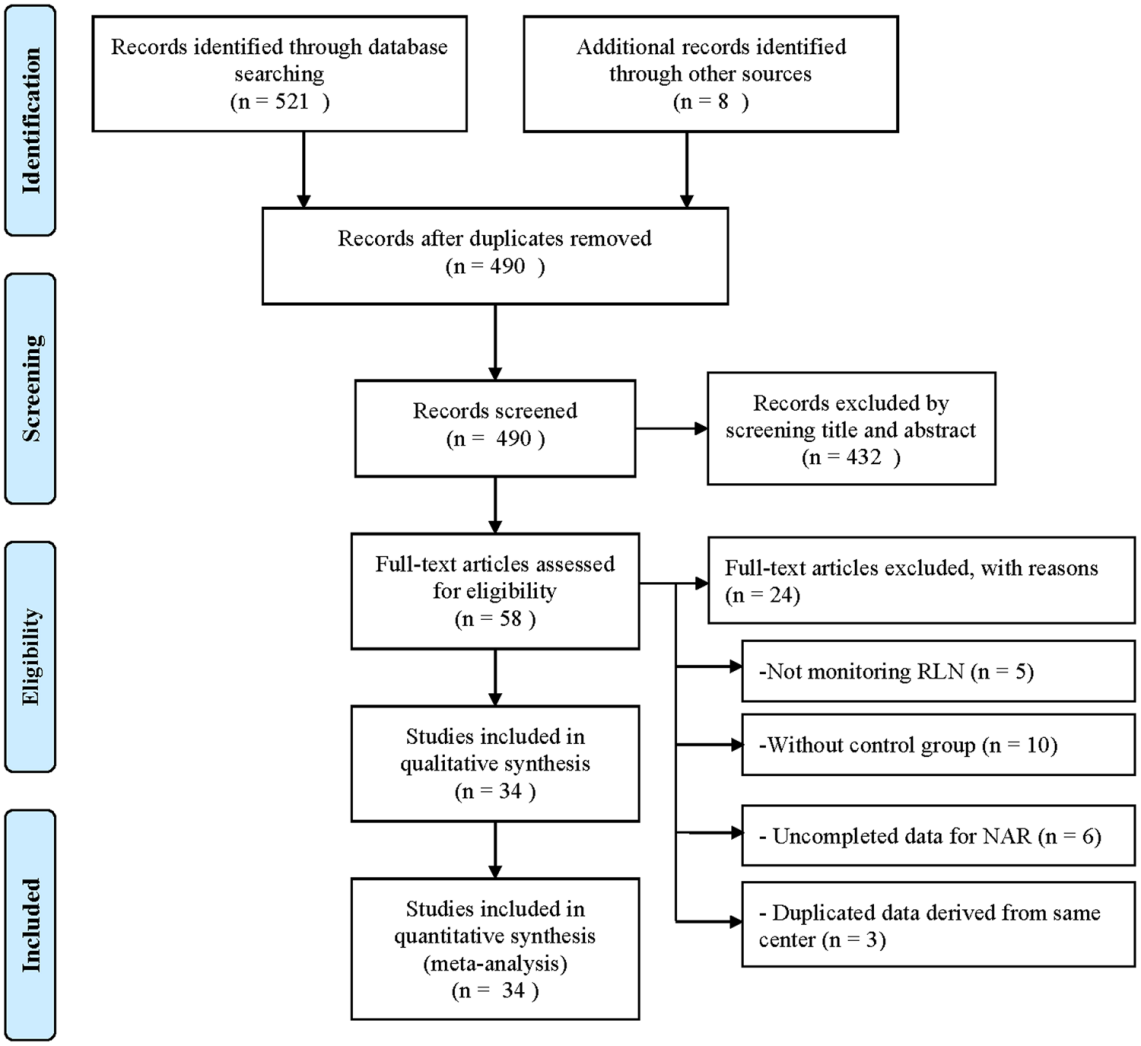
Flow diagram of study selection according to PRISMA statement^34^. Abbreviation: RLN = recurrent laryngeal nerve; NAR = nerves at risk.

A total of 529 articles were identified through literature research. After duplicates were removed, 490 articles were included for screening. Among these, 58 eligible full-text articles were selected after carefully checking the titles and abstracts. However, we excluded 24 from these 58 articles because of monitoring other nerve (5 articles), no control group (10 articles), incomplete data for NAR (6 articles), or duplicated data derived from same center (3 articles) (Fig. 1)^34^. Finally, 34 articles were included for systematical review and meta-analysis^2–6,8,10,11,14,15,21–26,35–52^. Details on first author, year of publication, title, location, study type, allocation method, study duration, device used, outcome measurement and definition of permanent injury were recorded (Table 1). There were 3 randomized controlled trials^40,42,52^ and 31 non-randomized trials^2–6,8,10,11,14,15,21–26,35–39,41,43–51^ included in our study. The number of case and control groups and the event of total, transient and permanent RLN injury were also recorded (Appendix Table 1). The NOS of included research studies ranged from 6 to 9, suggesting an acceptable quality of the studies.

**Table 1:** Main characteristic of included studies.

Author	Year	Location	Study type	Allocation method	Study duration	Device used	Outcome measurement	Permanent injury definition
Brauckhoff^51^	2002	Germany	NCT	Non-IONM:1995–1997; IONM:1998–2001	1995–2001	NS	Laryngoscopy	NS
Thomusch^2^	2002	Germany	NCT	NS	January 1 - December 31, 1998	Neurosign 100	Laryngoscopy	6 months
Dralle^5^	2004	Germany	NCT	Choice of surgeon and Device availability	January 1, 1998 - January 15, 2001	Neurosign 100	Laryngoscopy	6 months
Robertson^3^	2004	USA	NCT	NS	April 1999 - December 2002	NIM-2.0	Laryngoscopy	NS
Yarbrough^4^	2004	USA	NCT	Non-IONM:1998–2000; IONM:2000–2003	October 1998 - January 2003	NS	Laryngoscopy	NS
Witt^35^	2005	USA	NCT	NS	1998–2003	NIM	Laryngoscopy	12 months
Chan^6^	2006	Hong Kong	NCT	Choice of surgeon and Device availability	January 2002 - August 2005	Neurosign 100	Laryngoscopy	12 months
Netto^36^	2007	Brazil	NCT	IONM: November 2003 - January 2006; Non-IONM: May 2003 - September 2003	November 2003 and January 2006	NIM-2.0	Laryngoscopy	3 months
Shindo^37^	2007	USA	NCT	Non-IONM:1998–2002; IONM:2002–2005	1998–2005	NS	Laryngoscopy	6 months
Terris^38^	2007	USA	NCT	NS	January of 2004 - November of 2006	NIM-2.0	Laryngoscopy	6 months
Atallah^39^	2009	France	NCT	Non-IONM: November 2003-December 2005; IONM: December 2005-July 2007	November 2003 - July 2007	NIM-2.0	Laryngoscopy	12 months
Barczynski^52^	2009	Poland	RCT	Randomized	January 2006 - June 2007	Neurosign 100	Laryngoscopy	12 months
Dionigi^40^	2009	Italy	RCT	Randomized	2007	NIM-2.0	Laryngoscopy	NS
Frattini^41^	2010	Italy	NCT	NS	NS	NIM-2.0	Laryngoscopy	12 months
Sari^42^	2010	Turkey	RCT	Randomized	September 2007 - September 2009	NIM	Laryngoscopy	12 months
Barczynski^43^	2011	Poland	NCT	Non-IONM:2003–2004;IONM:2005–2009	January 2003 - June 2009	Neurosign 100 NIM-2.0	Laryngoscopy	12 months
Alesina^8^	2012	Germany	NCT	NS	November 1999 - April 2011	Neurosign NIM-3.0	Laryngoscopy	6 months
Gremillion^44^	2012	USA	NCT	NS	2007–2010	NS	NS	NS
Chuang^45^	2013	Taiwan	NCT	NS	2001–2010	NIM	NS	NS
Prokopakis^46^	2013	Greece	NCT	Incidentally	2004–2011	NIM	Laryngoscopy	4 months
Alesina^47^	2014	Germany	NCT	NS	January 2005 - December 2012	Neurosign NIM-3.0	Laryngoscopy	6 months
Barczynski^14^	2014	Poland	NCT	NS	1993–2012	Neurosign 100 NIM 2.0/3.0	Laryngoscopy	12 months
De Falco^48^	2014	Italy	NCT	Non-IONM:1 October 2009–31 July 2010; IONM:1 September 2010–31 October 2011	1 October 2009–31 October 2011	NS	Laryngoscopy	6 months
Sanguinetti^49^	2014	Italy	NCT	NS	2012	NS	Laryngoscopy	NS
de Danschutter^50^	2015	Netherlands	NCT	Non-IONM: September 2009 - July 2010; IONM: July 2010 - October 2012	September 2009 - October 2012	NIM-3.0	Laryngoscopy	12 months
Page^15^	2015	France	NCT	Non-IONM:2001–2004;IONM: 2005–2010	January 2001 - January 2010	Neurosign 400	Laryngoscopy	12 months
Anuwong^10^	2016	Italy	NCT	Non-IONM: 1995–2005; IONM: 2006–2013	January 2002 - December 2014	NIM-2.0/3.0	Laryngoscopy	12 months
Brajcich^21^	2016	USA	NCT	Non-IONM:2009–2013; IONM:2013–2015	2009–2015	NIM-3.0	Laryngoscopy	12 months
Calo^22^	2016	Italy	NCT	Device availability	June 2007 - December 2013	NIM-2.0/3.0	Laryngoscopy	12 months
Hei^11^	2016	China	NCT	Randomly allocated	October 2009 - August 2011	NIM-2.0	Laryngoscopy	6 months
Lv^23^	2016	China	NCT	Non-IONM:2010–2012; IONM:2012–2014	January 2010 - January 2014	NIM-3.0	Laryngoscopy	6 months
Vasileiadis^24^	2016	Greece	NCT	Device availability	January 2002 - December 2012	NIM-2.0	Laryngoscopy	12 months
Xie^25^	2016	China	NCT	NS	January 2012 - September 2014	NIM-3.0	Laryngoscopy	6 months
Kai^26^	2017	China	NCT	NS	1 January 2013–30 June 2016	NIM-3.0	Laryngoscopy	6 months

Abbreviation: NCT, non-randomized comparative trial; RCT, randomized controlled trial; NS, not stated.

There were mainly three types of devices used in the studies, including Neurosign 100, NIM 2.0 and NIM 3.0. Except for some papers that had not declared the outcome measurement, the majority of the included studies applied laryngoscopy to check the movement of vocal cords. 29 studies were conducted under general operation, 3 studies were based on minimally invasive thyroidectomy, and the other 2 studies were based on total endoscopic thyroidectomy (Appendix Table 2). Most of the studies were single-center trials except for 2 multi-center trials. In subgroup analysis, we focused on major influence factors, such as bilateral operation, malignancy, reoperation and operation volume (Appendix Table 3).

### Publication bias and sensitivity analysis

**Figure 2:**
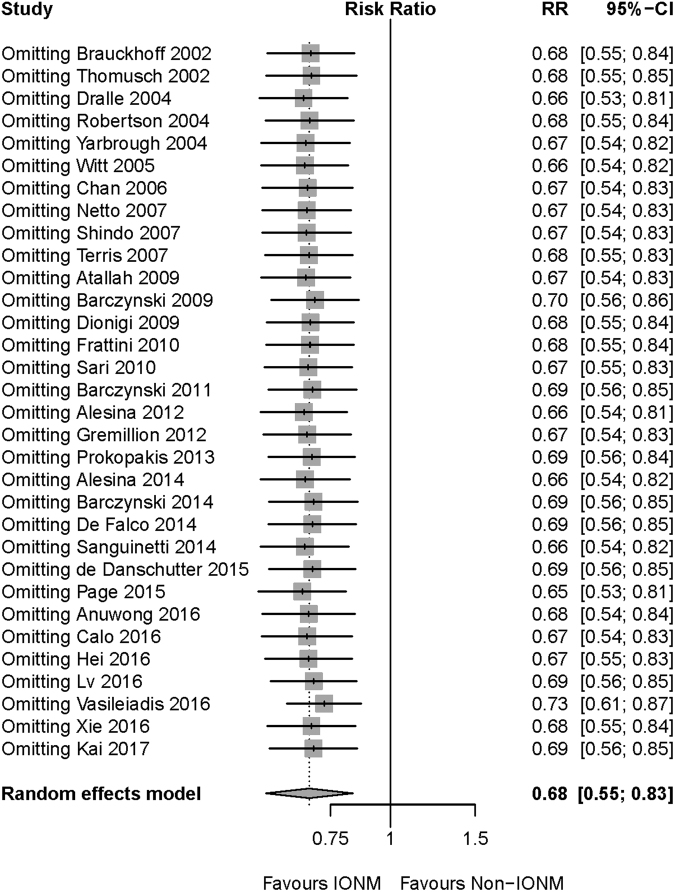
Sensitivity analysis of total RLN injury. After omitting any of the included studies, the meta-analysis result of total RLN injury was still robust. Abbreviation: IONM = intraoperative nerve monitoring; RLN = recurrent laryngeal nerve.

**Figure 3:**
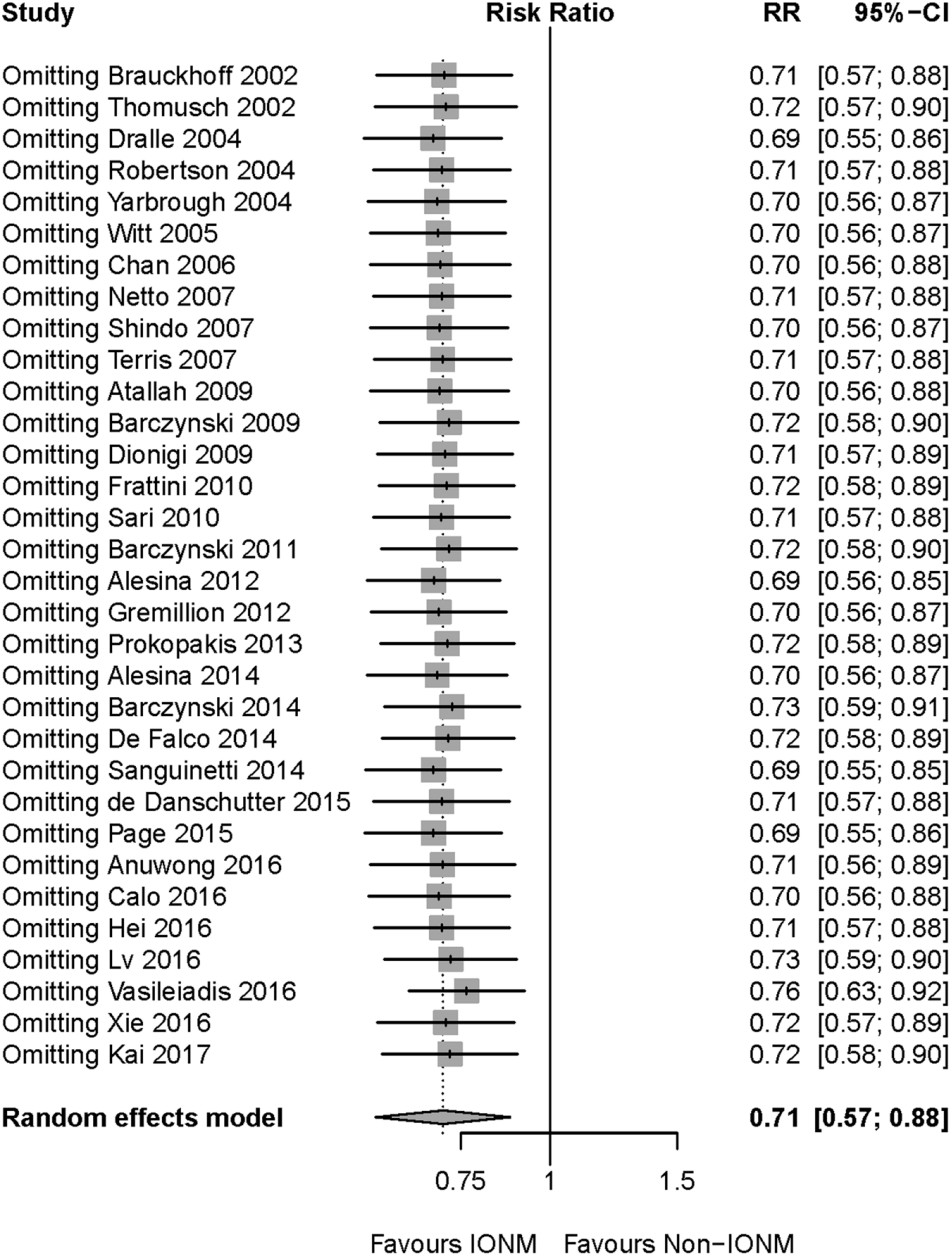
Sensitivity analysis of transient RLN injury. After omitting any of the included studies, the meta-analysis result of transient RLN injury was still robust. Abbreviation: IONM = intraoperative nerve monitoring; RLN = recurrent laryngeal nerve.

**Figure 4:**
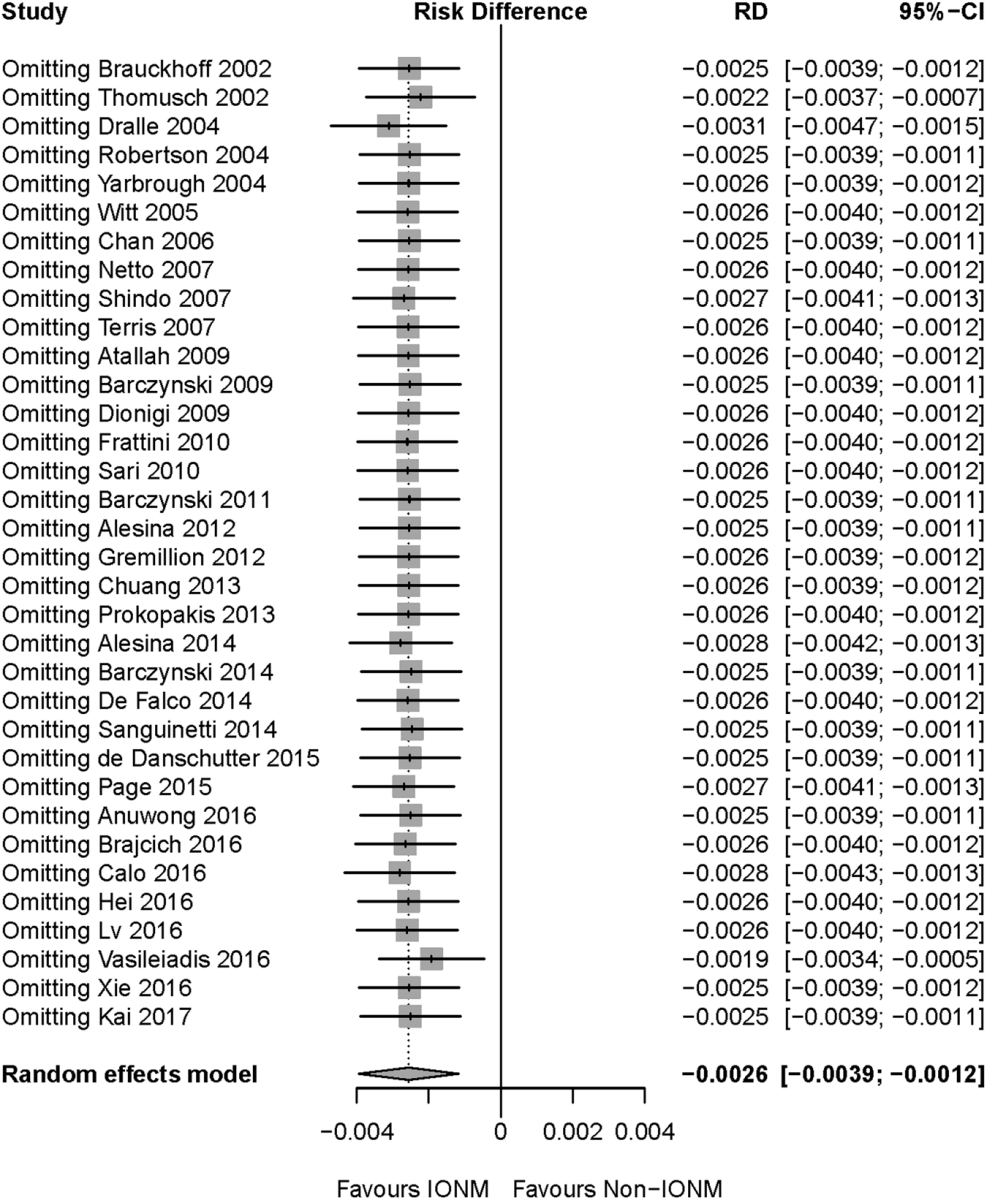
Sensitivity analysis of permanent RLN injury. After omitting any of the included studies, the meta-analysis result of permanent RLN injury was still robust. Abbreviation: IONM = intraoperative nerve monitoring; RLN = recurrent laryngeal nerve.

Begg’s and Egger’s test were conducted to assess the publication bias in this meta-analysis. None of the tests was statistically significant in total injury (Begg’s p = 0.97 and Egger’s p = 0.14), transient injury (Begg’s p = 0.79 and Egger’s p = 0.15) and permanent injury (Begg’s p = 0.012 and Egger’s p = 0.19). Meta-analysis was recalculated after omitting every record included in the analysis to check the sensitivity of our overall results. As shown in Figs 2, 3 and 4, the results of total, transient and permanent injury were robust after omitting any record.

### Overall meta-analysis

**Figure 5:**
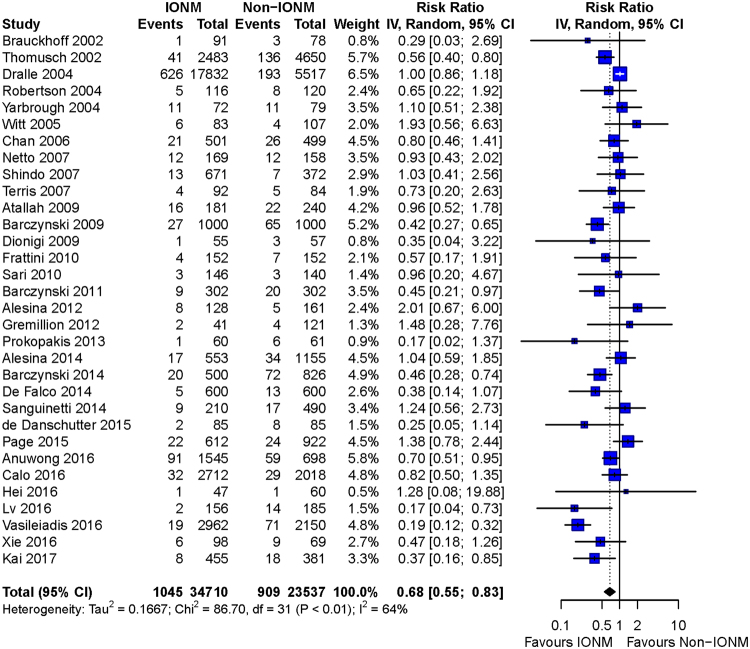
Forest plot of total RLN injury. Less total RLN injuries were found in IONM group than in non-IONM group by pooled analysis (p = 0.0002). Abbreviation: IONM = intraoperative nerve monitoring; RLN = recurrent laryngeal nerve.

**Figure 6:**
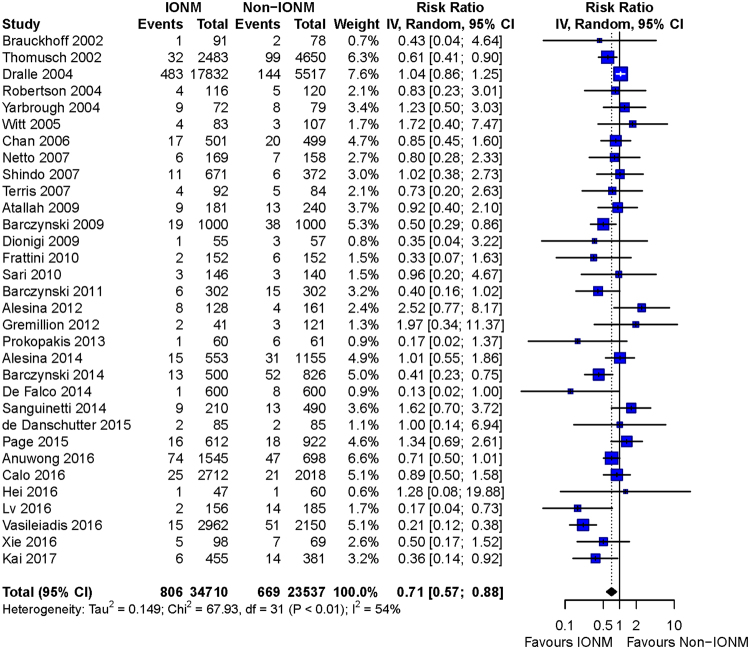
Forest plot of transient RLN injury. Meta-analysis showed a preventive effect of IONM on transient RLN injury (p = 0.0017). Abbreviation: IONM = intraoperative nerve monitoring; RLN = recurrent laryngeal nerve.

**Figure 7:**
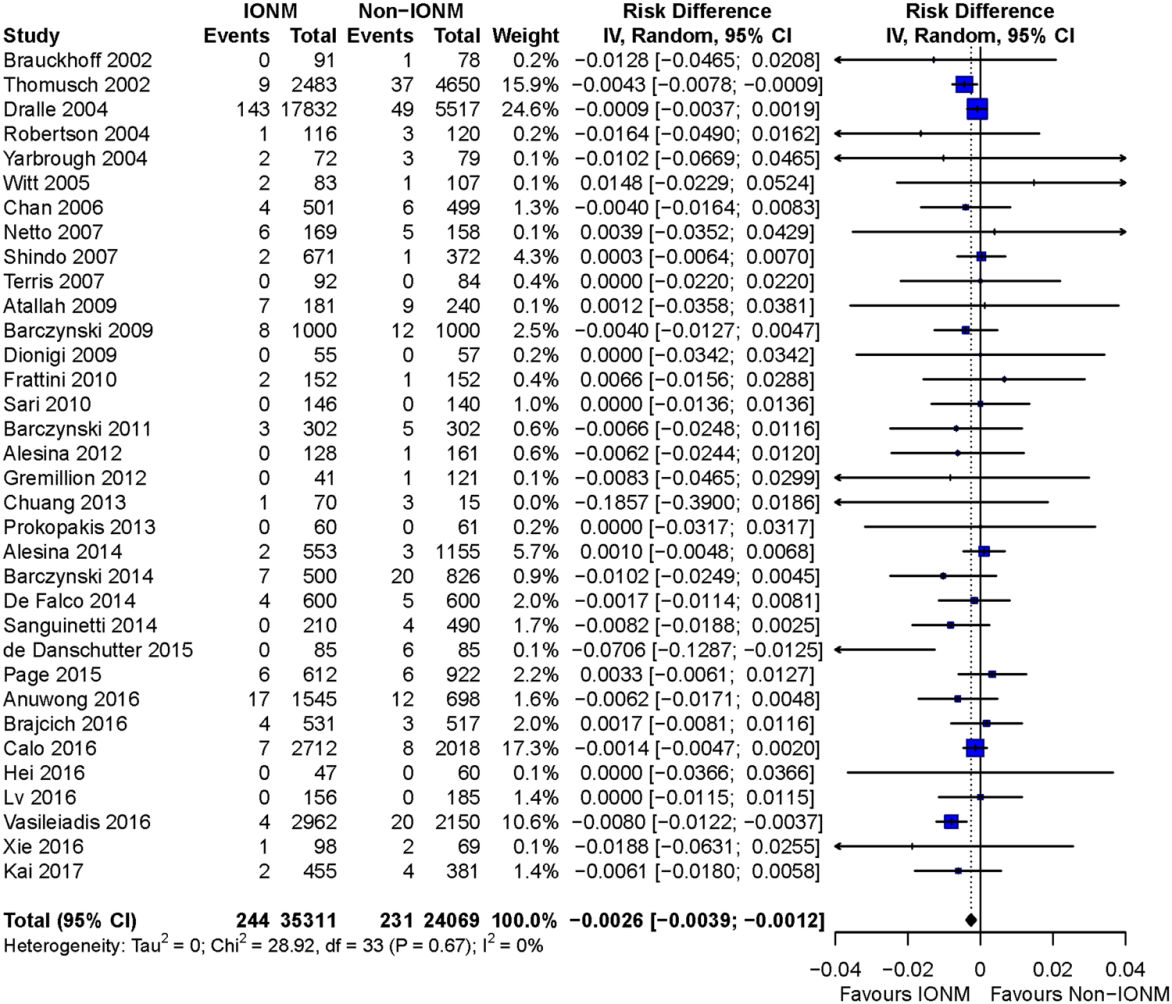
Forest plot of permanent RLN injury. Meta-analysis demonstrated a 0.26% reduction of permanent RLN injury with IONM (p = 0.0003). Abbreviation: IONM = intraoperative nerve monitoring; RLN = recurrent laryngeal nerve.

Overall meta-analysis found a significant decrease of total RLN injury (RR = 0.68, 95%CI: 0.55 to 0.83; p = 0.0002), transient injury (RR = 0.71, 95%CI: 0.57 to 0.88; p = 0.0017), and permanent injury (RD = −0.0026, 95%CI: −0.0039 to −0.0012; p = 0.0003) by IONM (Figs 5, 6 and 7).

### Subgroup analysis for bilateral operation

A total of 11 studies were included in the analysis, of which 2 studies were without transient injury data. Subgroup analysis demonstrated IONM group had a lower incidence of total, transient and permanent injury than non-IONM group. (IONM group: total injury 1.46%, transient injury 1.07%, permanent injury 0.50%; non-IONM group: total injury 3.01%, transient injury 2.12%, permanent injury 0.75%).

### Subgroup analysis for malignancy operation

A total of 8 studies were included in the analysis. Data showed that IONM had protective effects in total injury and in transient injury. (Total injury: RR = 0.61, 95% CI: 0.42 to 0.91; p = 0.02; Transient injury RR = 0.61, 95% CI: 0.38 to 0.97; p = 0.04) However, IONM had no significant protective effect in permanent injury. (RD = −0.0009, 95%CI: −0.0063 to 0.0044; p = 0.73).

### Subgroup analysis for reoperation group

A total of 9 studies were included in the analysis. The rate of total (RR = 0.64, 95% CI: 0.33–1.24; p = 0.1843), transient (RD = −0.0113, 95% CI: −0.0502 to 0.0276; p = 0.5687) or permanent injury (RD = −0.0082, 95% CI: −0.0176 to 0.0013; p = 0.0906) had no difference between IONM and non-IONM in the reoperation subgroup.

### Subgroup analysis of operation volume

To evaluate the effect of operation volume, we used the criteria of 300 NARs as the cut off to divide operation volume, as consistent with relevant studies^5,53^. IONM could reduce the incidence of total (RR = 0.78, 95%CI: 0.63 to 0.97; p = 0.0271) and transient (RR = 0.79, 95%CI: 0.63 to 0.99; p = 0.0454) injury in the group with operation volume < 300 NARs per year. Analysis also found a preventive effect of IONM on total and permanent injury in the group with operation volume > 300 NARs per year. (p = 0.0292 and p = 0.0247, correspondingly).

## Discussion

IONM had received widespread acceptance in the field of thyroid surgery for its role in RLN identification. However, the protective effect of IONM on RLN injury remained controversial. Zheng *et al*. proposed that IONM could reduce total and transient RLN injury in thyroidectomy^13^, but the results of transient injury had been proven to be hypersensitive^13^. Lombardi *et al*. gave a negative conclusion about the protective effects of IONM on permanent injury^18^. Wong *et al*. demonstrated that high-risk subpopulations might benefit from IONM, especially in reoperation and malignancy subgroups^17^. Several large studies were recently published in the past year; Some studies found preventive effects of IONM on total and transient injury^23,24,26^, while others declared an insignificant result on this topic^10,11,22,25^. To identify the protective effect of IONM on RLN function and explore more subgroups which could benefit from IONM, we conducted a meta-analysis that included the most recent developments in this field. Additionally, our meta-analysis was the first to investigate the protective effect of IONM on the topic of bilateral operation and operation volume and revealed the preventive effect of IONM on permanent injury in these subgroups.

Our meta-analysis found preventive effects of IONM on total, transient and permanent RLN injury. The result was still robust after sensitivity analysis. Previous meta-analyses found a decrease of total and transient injury in IONM cases, but for permanent injury the result was not statistically significant^12,13^. The negative results might result from small data sample size (NARs less than 40,000). A total of 59,380 NAR involved in this analysis was enough to reveal IONM’s effect on permanent injury. Lombardi *et al*. mentioned the influence of permanent injury definition and measurement^18^. In this study, both the permanent injury definition (over 6 months) and laryngoscopy measurement were included and did not influence the final results (Appendix Table 3). Based on existing results, we recommended IONM as a routine measurement to be used in thyroidectomy.

In subgroup analyses, the study revealed preventive effects of IONM on total and transient RLN injury in bilateral operation and malignancy operation subgroups. However, for permanent injury, a similar effect was only found in bilateral operation cases. Bilateral thyroidectomy requires relative large operation extent and long operation duration. Previous studies revealed RLN injury incidence in bilateral operation was 1.2–2.3%^2,5^. In this study, pooled incidences of total, transient and permanent injury in bilateral operation were 2.3%, 1.6% and 0.61%. Our results supported the recommendation of IONM application in bilateral operation. Above all, bilateral operation might induce bilateral RLN injury, which could lead to airway obstruction. To avoid this fatal complication, someone proposed routine IONM application in bilateral operation and a staged thyroidectomy would be needed for cases with intraoperative RLN signal loss^19^.

Previous meta-analyses only showed positive results in transient injury for malignancy operation. Our analysis included 3 new studies^11,21,25^ and 1 study with updated data^22^. The results demonstrated a statistically significant decrease of total and transient injury in malignancy operation cases with IONM. Malignancy operation might contain potential risk of malignancy invasion of RLN and lymph node dissection, which increase the risk of RLN injury. IONM might reduce the risk by real-time monitor of RLN function.

Reoperation of the thyroid has higher risks than normal thyroidectomy, for local conglutination and anatomical changes can result in RLN injury. Local conglutination makes the identification of RLN difficult and the anatomical position of RLN might change due to a previous operation. Wong *et al*. found a decreased incidence of total injury in the reoperation group with IONM. However, our study demonstrated a negative result in the reoperation group with IONM, with 2 new studies^21,45^ and 1 study with updated data^22^ included. The effect of IONM in this subgroup still needs to be identified. Further large size randomized trials need to be conducted to clarify this debate.

The impact of operation volume was also emphasized in several studies. In a German retrospective multicenter trial, a large operation volume medical center had a lower rate of permanent RLN injury, and thus IONM might be more helpful in small operation volume centers than in large operation volume centers^5^. However, in our subgroup analysis, we found IONM could help both small and large operation volume medical centers. IONM reduced total and transient RLN injury in small operation volume centers, and decreased total and permanent RLN injury in large operation volume centers. With the aid of IONM, inexperienced surgeons could avoid hazardous surgical situations near or on the RLN with real-time identification of the RLN^54^.

There were several limitations in our study. First, there were still several studies didn’t declare the outcome measurement or follow up for enough duration. The robust test had been passed including all the studies with laryngoscopy measurement and more than 6 months of follow-up. Second, there still existed confounders and heterogeneity in our analysis. Some studies included cases that involved intentional RLN transection. The influence of lymph node dissection could also not be eliminated. The sensitivity analysis, subgroup analysis, and random-effects model was applied to address this.

## Conclusions

This meta-analysis indicated that IONM could reduce the incidence of total, transient and permanent RLN injury compared with conventional visual identification. We also recommended IONM in bilateral operations and malignancy operations. IONM could help surgeons perform a better thyroidectomy regardless of the medical center’s operation volume. The benefit of IONM in reoperation cases needs to be further explored. We hope our findings can be assessed by large prospective, randomized clinical trials in the future with a standardized IONM application and outcome measurement.

## Electronic supplementary material

Appendix Tables
