# Supplementary material for: Protective Effects of Intraoperative Nerve Monitoring (IONM) for Recurrent Laryngeal Nerve Injury in Thyroidectomy: Meta-analysis
Source: Sci Rep. 2018 May 17;8:7761. doi: 10.1038/s41598-018-26219-5 (PMC5958090; doi:10.1038/s41598-018-26219-5)
Supplement: Supplementary file 1 — Appendix Tables [file 41598_2018_26219_MOESM1_ESM.pdf]

Protective Effects of Intraoperative Nerve Monitoring (IONM) for Recurrent Laryngeal Nerve Injury in Thyroidectomy: Meta-analysis

Binglong Bai[1]; Wuzhen Chen[2*]

1. Department of General Surgery (Thyroid Center), Second Affiliated Hospital, School of Medicine, Zhejiang University, Hangzhou, Zhejiang, P. R. China.
2. Department of Surgical Oncology, Second Affiliated Hospital, School of Medicine, Zhejiang University, Hangzhou, Zhejiang, P. R. China.

**Correspondence Author*
Wuzhen Chen
Department of Surgical Oncology, Second Affiliated Hospital, Zhejiang University School of Medicine,
Hangzhou 310009, China.
E-mail: chenwuzhen@zju.edu.cn

Appendix Table 1. Incidence of total, transient and permanent RLN injury

Study	Patients, NAR		Total RLN injury		Transient RLN injury		Permanent RLN injury	
	IONM	Non-IONM	IONM	Non-IONM	IONM	Non-IONM	IONM	Non-IONM
Brauckhoff[50]	91	78	1	3	1	2	0	1
Thomusch[2]	2483	4650	41	136	32	99	9	37
Dralle[5]	17832	5517	626	193	483	144	143	49
Robertson[3]	116	120	5	8	4	5	1	3
Yarbrough[4]	72	79	11	11	9	8	2	3
Witt[34]	83	107	6	4	4	3	2	1
Chan[6]	501	499	21	26	17	20	4	6
Netto[35]	169	158	12	12	6	7	6	5
Shindo[36]	671	372	13	7	11	6	2	1
Terris[37]	92	84	4	5	4	5	0	0
Atallah[38]	181	240	16	22	9	13	7	9
Barczynski[51]	1000	1000	27	65	19	38	8	12
Dionigi[39]	55	57	1	3	1	3	0	0
Frattini[40]	152	152	4	7	2	6	2	1
Sari[41]	146	140	3	3	3	3	0	0
Barczynski[42]	302	302	9	20	6	15	3	5
Alesina[8]	128	161	8	5	8	4	0	1
Gremillion[43]	41	121	2	4	2	3	0	1
Chuang[44]	70	15	NS*	NS*	NS#	NS#	1	3
Prokopakis[45]	60	61	1	6	1	6	0	0
Alesina[46]	553	1155	17	34	15	31	2	3
Barczynski[14]	500	826	20	72	13	52	7	20
De Falco[47]	600	600	5	13	1	8	4	5
Sanguinetti[48]	210	490	9	17	9	13	0	4
de Danschutter[49]	85	85	2	8	2	2	0	6
Page[15]	612	922	22	24	16	18	6	6
Anuwong[10]	1545	698	91	59	74	47	17	12
Brajcich[21]	531	517	NS*	NS*	NS#	NS#	4	3
Calo[22]	2712	2018	32	29	25	21	7	8
Hei[11]	47	60	1	1	1	1	0	0
Lv[23]	156	185	2	14	2	14	0	0
Vasileiadis[24]	2962	2150	19	71	15	51	4	20
Xie[25]	98	69	6	9	5	7	1	2
Kai[26]	455	381	8	18	6	14	2	4
Total	35311	24069	1045	909	806	669	244	231

Incidence	-	-	3.01%	3.86%	2.32%	2.84%	0.69%	0.96%

Abbreviation: NS, not stated.

* Total RLN injury referred to all postoperative RLN injury, including transient and permanent injury. The total RLN injury in studies, without data of transient injury, were also considered as NS.

Appendix Table 2. Details about factors of concern, population type, multicenter or single center and NOS score.

Author	Factors of concern			Operation type*	Multicenter or single center	Newcastle-Ottawa Scale		
	Bilateral procedure	Re-operation	Mali-gnancy			Selec-tion	Compar-ability	Outcome
Brauckhoff[50]				Open	Single center	4	1	2
Thomusch[2]	√			Open	Multicenter	4	1	3
Dralle[5]	√	√	√	Open	Multicenter	4	1	3
Robertson[3]				Open	Single center	4	1	2
Yarbrough[4]		√		Open	Single center	4	1	2
Witt[34]				Open	Single center	4	0	3
Chan[6]		√	√	Open	Single center	4	2	3
Netto[35]				Open	Single center	4	2	2
Shindo[36]				Open	Single center	4	1	2
Terris[37]				Endoscopic	Single center	4	1	3
Atallah[38]				Open	Single center	4	1	3
Barczynski[51]				Open	Single center	4	2	3
Dionigi[39]				Endoscopic	Single center	4	2	2
Frattini[40]	√		√	Open	Single center	4	2	3
Sari[41]				Open	Single center	4	2	3
Barczynski[42]	√		√	Open	Single center	4	2	3
Alesina[8]		√		Open	Single center	4	1	3
Gremillion[43]				Open	Single center	4	1	1

Chuang[44]		√		Open	Single center	4	1	1
Prokopakis[45]		√		Open	Single center	4	1	2
Alesina[46]				Open	Single center	4	2	3
Barczynski[14]		√		Open	Single center	4	0	3
De Falco[47]	√			Open	Single center	4	1	3
Sanguinetti[48]	√			Open	Single center	4	0	2
de Danschutter[49]				Open	Single center	4	2	3
Page[15]	√			Open	Single center	4	1	3
Anuwong[10]				Open	Single center	4	0	3
Brajcich[21]	√	√	√	Open	Single center	4	1	3
Calo[22]	√	√	√	Open	Single center	4	2	3
Hei[11]			√	Endoscopic	Single center	4	1	3
Lv[23]				Endoscopic	Single center	4	1	3
Vasileiadis[24]	√			Open	Single center	4	2	3
Xie[25]			√	Endoscopic	Single center	4	2	3
Kai[26]				Open	Single center	4	2	3

* Operation type was divided into two type: open operation and endoscopic surgery (minimally invasive surgery and total endoscopic surgery).

Appendix Table 3. Meta-analysis of subgroups based on factors of concern.

Subgroups		IONM		Non-IONM		RR [95% CI] or RD [95% CI]	p value	Number of studies
		Events	overall NAR	Events	overall NAR			
Bilateral procedure								
	Total injury	157	10746	351	11667	0.57 [0.37, 0.87]	0.0086	9
	Transient injury	115	10746	247	11667	0.57 [0.36, 0.91]	0.0182	9
	Permanent injury	115	22817	123	16502	0.68 [0.46, 0.99]	0.0433	11
Reoperation								
	Total injury	48	908	104	1203	0.64 [0.33, 1.24]	0.1843	7
	Transient injury	37	908	75	1203	-0.0113 [-0.0502, 0.0276]*	0.5687	7
	Permanent injury	44	1902	42	1565	-0.0082 [-0.0176, 0.0013]*	0.0906	9
Malignancy								
	Total injury	41	1579	57	1307	0.61 [0.41, 0.91]	0.0163	6
	Transient injury	31	1579	44	1307	0.61 [0.38, 0.97]	0.0355	6
	Permanent injury	34	2837	20	1824	-0.0008 [-0.0061, 0.0046]*	0.7801	8
Operation sites per year < 300								
	Total injury	272	6463	353	6657	0.78 [0.63, 0.97]	0.0271	20
	Transient injury	209	6463	265	6657	0.79 [0.63, 0.99]	0.0454	20
	Permanent injury	68	7064	94	7189	-0.0012 [-0.0041, 0.0016]*	0.4003	22
Operation sites per year ≥ 300								
	Total injury	92	7484	195	6258	0.49 [0.26, 0.93]	0.0292	5
	Transient injury	69	7484	131	6258	0.52 [0.25, 1.12]	0.0962	5

	Permanent injury	23	7484	49	6258	0.46 [0.23, 0.91]	0.0247	5
Permanent injury definition >6 months and laryngoscopy as measurement								
	Permanent injury	232	33756	210	22518	-0.0025[-0.0040, -0.0011]*	0.0005	24

* Data was presented as RD [95% CI] because of studies without any event in either IONM or non-IONM.
